# Lipoprotein(a) in nephrological patients

**DOI:** 10.1007/s11789-017-0086-z

**Published:** 2017-02-08

**Authors:** Bernd Hohenstein

**Affiliations:** 1 Nephrological Center Villingen-Schwenningen, Albert-Schweitzer-Str. 6, 78052 Villingen-Schwenningen, Germany; 2 Faculty of Medicine Carl Gustav Carus, Fetscherstraße 74, 01307 Dresden, Germany

**Keywords:** Lipoprotein(a), Kidney disease, Cardiovascular risk, Lipids

## Abstract

In contrast to existing EAS/ESC guidelines on the management of lipid disorders, current recommendations from nephrological societies are very conservative and restrictive with respect to any escalation of lipid lowering/statin therapy. Furthermore, lipoprotein(a) (Lp(a)) – an established cardiovascular risk factor – has not even been mentioned. While a number of retrospective and prospective studies suggested that Lp(a) has relevant predictive value and might have – at least in stage-3 chronic kidney disease (CKD) – the same negative effects if draged along in non-CKD patients, there is no guidance on diagnostic or therapeutic procedures. The persistent lack of recognition automatically leads to therapeutic nihilism, which might pose a number of relatively young patients to a significantly increased risk for adverse cardiovascular events. Further evaluation of Lp(a) in CKD is very important to provide appropriate treatment to patients with high Lp(a) levels, even in the presence of CKD.

## Current guidance and controversies in lipidology versus nephrology

With the introduction of new drugs targeting proprotein convertase subtilisin kexine type 9 (PCSK9), the effective, target-oriented treatment of lipid disorders received a new perspective [1, 2]. While the IMPROVE-IT trial clearly demonstrated that “even lower” is “even better”, thereby proving the concept of targeting LDL-C values of 70 mg/dL (1.8 mmol/L) or lower in cardiovascular high-risk patients [3], the new drugs will offer the chance of reaching this target in the vast majority of patients [4].

Recent guidelines from the European Societies of Cardiology (ESC) and Atherosclerosis (EAS) recommend – based on the overall cardiovascular risk – that patients with a moderately reduced eGFR of 60–30 ml/min/1.73 m^2^ should be classified as high-risk and with a eGFR below 30 ml/min/1.73 m^2^ as very high-risk patients [5]. While these guidelines recommend a strict to target reduction of LDL-C levels, current Kidney Disease Improving Global Outcomes (KDIGO) clinical practice guidelines for lipid management in chronic kidney disease (CKD) do not see evidence beyond the single-point evaluation of a patient’s lipid status, including total cholesterol, LDL-cholesterol, HDL-cholesterol and triglycerides, followed by a fire-and-forget strategy [6]. European best practice guidelines further restrict the LDL-C lowering treatment to patients with diabetes mellitus [7].

In contrast to ESC and EAS, KDIGO recommendations are mainly based on results from the three major interventional trials in patients on dialysis and suffering from CKD: 4D, AURORA and SHARP [8–10]. While these results suggest statin treatment as a primary prevention in patients with moderate and advanced CKD [10], statins have now proven efficacy for secondary prevention in dialysis patients [8, 9]. Results in dialysis patients are most likely related to the different pathophysiology developing during CKD, shifting the dominance of classical cardiovascular (CV) risk factors towards CKD related factors such as hyperphosphatemia, increased calcium-phosphate product, secondary hyperparathyroidism, and a lack of calcification inhibiting factors finally leading to more pronounced media sclerosis and vascular stiffness [11].

So far, existing nephrological guidelines do not even recommend the measurement of Lipoprotein (a) (Lp(a)).

## Lipoprotein (a) levels in kidney patients

Starting in the early 90s, a number of studies investigated the potential role of Lp(a) in CKD patients. As depicted by Kronenberg and colleagues, it is clear that Lp(a) levels start to rise with decreasing glomerular filtration rate (GFR) being fourfold higher in patients with nephrotic range proteinuria compared to healthy controls [12]. This is completely corrected in kidney transplant patients and partially reduced in patients on hemodialysis and to a lesser extent also in patients undergoing peritoneal dialysis.

From a pathophysiologic point of view, *in-vivo* studies demonstrated that patients with nephrotic syndrome have an increased Lp(a) synthesis rate, while hemodialysis patients have a steady production rate in presence of a prolonged residence time termed catabolic block [12].

## Relevance of high Lp(a) in CKD

Recently, the Chronic Renal Insufficiency Cohort (CRIC) study performed a systematic measurement of serum lipids and lipoproteins in a CKD cohort of 3939 patients [13]. They tried to detect factors being relevant for the progression of CKD, but failed to demonstrate any associations with lipid parameters and especially with Lp(a).

So far, most investigations focused on patients already undergoing dialysis treatment. More than ten years ago, the Choices for Healthy Outcomes in Caring for End-Stage Renal Disease (CHOICE) study prospectively included incident dialysis patients [14, 15]. In 864 out of 1041 a Lp(a) measurement was performed. The investigators aimed to answer the questions, whether small apo(a) size and/or high Lp(a) levels predict mortality or CV events in dialysis patients. While they found that small apo(a) size, but not a high Lp(a) level was predictive for mortality [14], they later published a second study stating that high Lp(a) levels as well as small apo(a) size can predict CV events in dialysis patients [15]. In the later study, patients with more than 22 kringle-type IV repeats and Lp(a) concentrations of more than 123 nmol/L (4th quartile) had a 1.73 fold risk for CV events (*p* < 0.0005). While experimental data in uremic mice pointed into the same direction [16], further confirming studies in CKD have not been performed until today.

More recently, investigators from Japan evaluated 904 patients with CKD out of 3508 patients undergoing a percutaneous coronary intervention (PCI) [17]. The comparison between high (*n* = 454) and low (*n* = 450) Lp(a) levels regarding all-cause death and acute coronary syndrome over a period of 4.7 years found a worse outcome in those with higher Lp(a) levels. Lp(a) seemed to be an independent predictor of adverse outcomes in CKD patients following PCI.

Finally, Kollerits and colleagues performed a post-hoc analysis of patients participating in the 4D study which included type 2 diabetics undergoing hemodialysis either treated with 20 mg of atorvastatin or placebo [18]. 1233 out of 1255 initial samples were available and measurement of Lp(a) was performed at baseline and after 6 months. The authors divided patients according to their Lp(a) levels as well as low and high molecular weight isoforms into quartiles. Increased Lp(a) concentrations were associated with all-cause mortality, but this effect was mainly driven by infections. This Lp(a) related effect was especially prominent in younger patients (<66 years of age), which also had a higher risk of fatal stroke (hazard ratio 1.54; *P* = 0.03).

At least in part, these studies confirmed two earlier findings published by Kronenberg and colleagues investigating the relevance of Lp(a) for the development of coronary artery disease and carotid atherosclerosis in end-stage renal disease patients more than 20 years ago [19, 20].

In both studies low molecular weight Lp(a) isoforms was associated with more severe atherosclerotic changes. While Lp(a) concentrations were not linked with CAD, they associated with carotid atherosclerosis and the number of affected vascular beds.

Of note, all these publications widely reflect patients having moderately increased Lp(a) levels below the currently accepted threshold of 120 nmol/L or 60 mg/dL indicating the necessity of lipoprotein apheresis according to the German Federal Joint Committee [21].

## Conclusions from existing literature

In summary, a number of prospective studies pointed towards a relevance of Lp(a) as relevant CV risk factor in CKD and dialysis patients predicting both, the development of atherosclerotic lesions and adverse outcomes. Even though CKD related vascular pathology becomes more relevant in advanced stages of CKD, it is very likely that Lp(a) – as in non-CKD individuals – significantly contributes to the development and progression of atherosclerotic lesions. Yet, it is unclear to which extent very high Lp(a) levels (above the 90% percentile) will influence CAD in CKD patients. Especially in moderate CKD, it seems to be important to carefully evaluate traditional risk factors including Lp(a) and take this into consideration and treatment decisions. Clearly, further studies in CKD patients are necessary, but momentary nihilism will not prevent any adverse event.

## Dealing with high Lp(a) in daily clinical practice

**Fig. 1:**
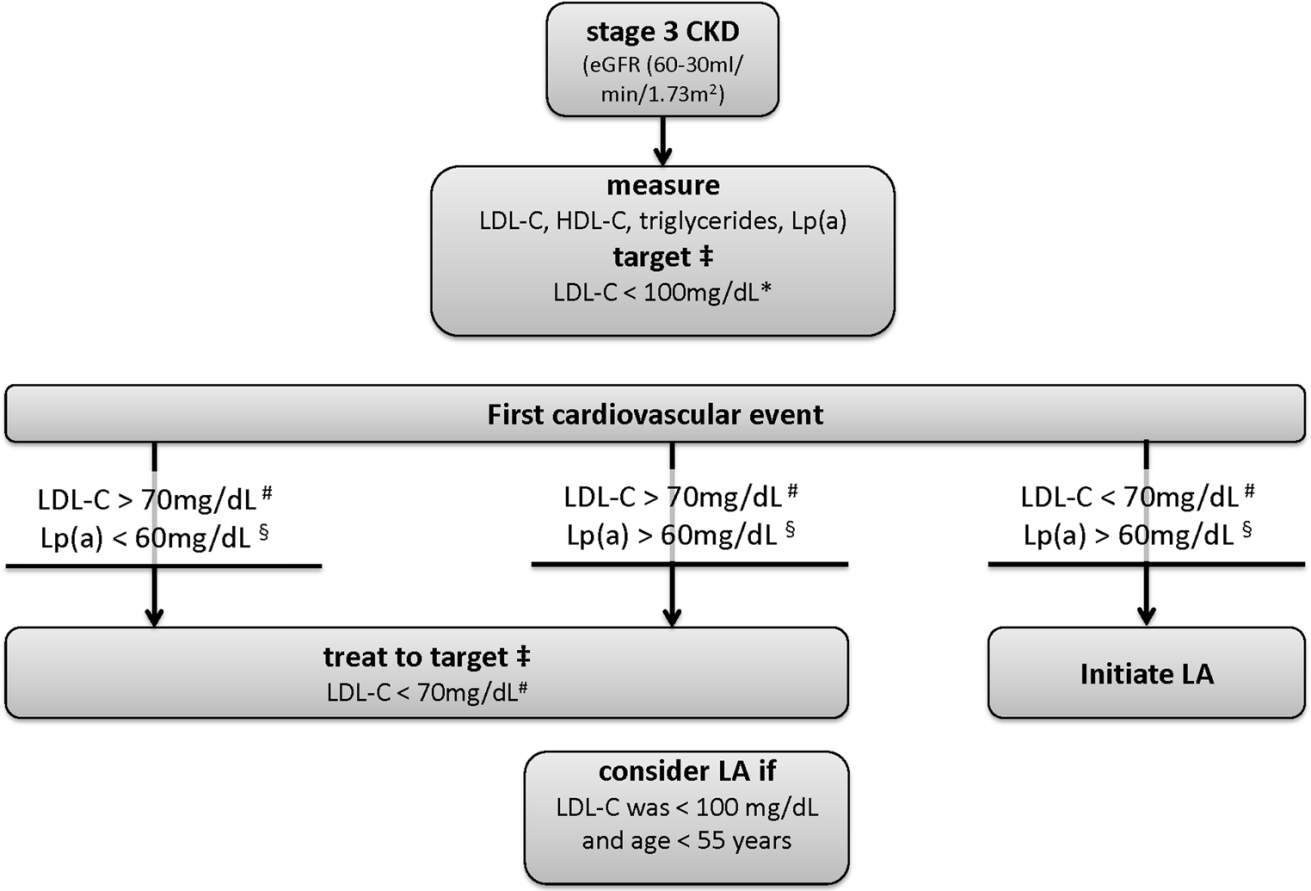
Proposed handling of lipid disorders in CKD incl. Lp(a). * 2.6 mmol/L; # 1.8 mmol/L; § 120 nmol/L; LA = lipoprotein apheresis; ‡ using atorvastatin, simvastatin/ezetemibe, rosuvastatin, or atorvastatin/ezetimibe, and/or PCSK9i

Especially in early stages of CKD, Lp(a) should be measured in every patient since the CKD-related increase will be limited in these patients and less controversies on the role of decreased eGFR will occur. CV events in CKD stage 3 patients at the age of 50–55 or below in presence of significantly elevated Lp(a) levels should not be primarily accepted as a consequence of CKD, but undergo intensive workup of all vascular beds (carotids, peripheral vessels, coronaries, aorta) and aggressive treatment. According to the accepted German guidelines [21], lipoprotein apheresis should be initiated in patients with progressive CV disease and Lp(a) levels of more than 60 mg/dL (or 120 nmol/L). Individualized decisions are necessary, even more carefully in advanced CKD. It is important to note that the relevance of Lp(a) in patients after kidney transplantation (KTx) is completely unclear. However, early manifestations of CV disease at an early stage of CKD might also guide the treatment decision after KTx, since CV disease is one of the leading causes for the loss of a functioning graft. An algorithm for the handling of lipid disorders including Lp(a) in CKD is proposed in Fig. 1.

## References

[1] Sabatine MS, Giugliano RP, Wiviott SD, Raal FJ, Blom DJ, Robinson J, Ballantyne CM, Somaratne R, Legg J, Wasserman SM, Scott R, Koren MJ, Stein EA, Open-Label Study of Long-Term Evaluation against LDLCI (2015). Efficacy and safety of evolocumab in reducing lipids and cardiovascular events. N Engl J Med.

[2] Robinson JG, Farnier M, Krempf M, Bergeron J, Luc G, Averna M, Stroes ES, Langslet G, Raal FJ, El Shahawy M, Koren MJ, Lepor NE, Lorenzato C, Pordy R, Chaudhari U, Kastelein JJ, OLT Investigators (2015). Efficacy and safety of alirocumab in reducing lipids and cardiovascular events. N Engl J Med.

[3] Cannon CP, Blazing MA, Giugliano RP, McCagg A, White JA, Theroux P, Darius H, Lewis BS, Ophuis TO, Jukema JW, De Ferrari GM, Ruzyllo W, De Lucca P, Im K, Bohula EA, Reist C, Wiviott SD, Tershakovec AM, Musliner TA, Braunwald E, Califf RM, IMPROVE-IT Investigators (2015). Ezetimibe added to statin therapy after acute coronary syndromes. N Engl J Med.

[4] Stroes E, Colquhoun D, Sullivan D, Civeira F, Rosenson RS, Watts GF, Bruckert E, Cho L, Dent R, Knusel B, Xue A, Scott R, Wasserman SM, Rocco M, GAUSS-2 Investigators (2014). Anti-PCSK9 antibody effectively lowers cholesterol in patients with statin intolerance: the GAUSS-2 randomized, placebo-controlled phase 3 clinical trial of evolocumab. J Am Coll Cardiol.

[5] Catapano AL, Graham I, De Backer G, Wiklund O, Chapman MJ, Drexel H, Hoes AW, Jennings CS, Landmesser U, Pedersen TR, Reiner Z, Riccardi G, Tokgozoglu L, Verschuren WM, Vlachopoulos C, Wood DA, Zamorano JL, Taskinen MR, Authors/Task Force (2016). 2016 ESC/EAS Guidelines for the Management of Dyslipidaemias: The Task Force for the Management of Dyslipidaemias of the European Society of Cardiology (ESC) and European Atherosclerosis Society (EAS)Developed with the special contribution of the European Assocciation for Cardiovascular Prevention & Rehabilitation (EACPR). Eur Heart J.

[6] KDIGO KLW Group (2013). KDIGO clinical practice guideline for lipid management in chronic kidney disease. Kidney Int Suppl.

[7] Guideline Development Group (2015). Clinical Practice Guideline on management of patients with diabetes and chronic kidney disease stage 3b or higher (eGFR 〈45 mL/min). Nephrol Dial Transplant.

[8] Wanner C, Krane V, Marz W, Olschewski M, Mann JF, Ruf G, Ritz E, German Diabetes and Dialysis Study Investigators (2005). Atorvastatin in patients with type 2 diabetes mellitus undergoing hemodialysis. N Engl J Med.

[9] Fellstrom BC, Jardine AG, Schmieder RE, Holdaas H, Bannister K, Beutler J, Chae DW, Chevaile A, Cobbe SM, Gronhagen-Riska C, De Lima JJ, Lins R, Mayer G, McMahon AW, Parving HH, Remuzzi G, Samuelsson O, Sonkodi S, Sci D, Suleymanlar G, Tsakiris D, Tesar V, Todorov V, Wiecek A, Wuthrich RP, Gottlow M, Johnsson E, Zannad F, AURORA Study Group (2009). Rosuvastatin and cardiovascular events in patients undergoing hemodialysis. N Engl J Med.

[10] Sharp Collaborative Group (2010). Study of Heart and Renal Protection (SHARP): randomized trial to assess the effects of lowering low-density lipoprotein cholesterol among 9,438 patients with chronic kidney disease. Am Heart J.

[11] Ketteler M, Rothe H, Kruger T, Biggar PH, Schlieper G (2011). Mechanisms and treatment of extraosseous calcification in chronic kidney disease. Nat Rev Nephrol.

[12] Kronenberg F (2014). Causes and consequences of lipoprotein(a) abnormalities in kidney disease. Clin Exp Nephrol.

[13] Rahman M, Yang W, Akkina S, Alper A, Anderson AH, Appel LJ, He J, Raj DS, Schelling J, Strauss L, Teal V, Rader DJ, CRIC Study Investigators (2014). Relation of serum lipids and lipoproteins with progression of CKD: The CRIC study. Clin J Am Soc Nephrol.

[14] Longenecker JC, Klag MJ, Marcovina SM, Powe NR, Fink NE, Giaculli F, Coresh J (2002). Small apolipoprotein(a) size predicts mortality in end-stage renal disease: The CHOICE study. Circulation.

[15] Longenecker JC, Klag MJ, Marcovina SM, Liu YM, Jaar BG, Powe NR, Fink NE, Levey AS, Coresh J (2005). High lipoprotein(a) levels and small apolipoprotein(a) size prospectively predict cardiovascular events in dialysis patients. J Am Soc Nephrol.

[16] Pedersen TX, McCormick SP, Tsimikas S, Bro S, Nielsen LB (2010). Lipoprotein(a) accelerates atherosclerosis in uremic mice. J Lipid Res.

[17] Konishi H, Miyauchi K, Tsuboi S, Ogita M, Naito R, Dohi T, Kasai T, Tamura H, Okazaki S, Isoda K, Daida H (2016). Plasma lipoprotein(a) predicts major cardiovascular events in patients with chronic kidney disease who undergo percutaneous coronary intervention. Int J Cardiol.

[18] Kollerits B, Drechsler C, Krane V, Lamina C, Marz W, Dieplinger H, Ritz E, Wanner C, Kronenberg F, German Diabetes and Dialysis Study Investigators (2016). Lipoprotein(a) concentrations, apolipoprotein(a) isoforms and clinical endpoints in haemodialysis patients with type 2 diabetes mellitus: results from the 4D Study. Nephrol Dial Transplant.

[19] Kronenberg F, Kathrein H, Konig P, Neyer U, Sturm W, Lhotta K, Grochenig E, Utermann G, Dieplinger H (1994). Apolipoprotein(a) phenotypes predict the risk for carotid atherosclerosis in patients with end-stage renal disease. Arterioscler. Thromb. Vasc. Biol..

[20] Koch M, Kutkuhn B, Trenkwalder E, Bach D, Grabensee B, Dieplinger H, Kronenberg F (1997). Apolipoprotein B, fibrinogen, HDL cholesterol, and apolipoprotein(a) phenotypes predict coronary artery disease in hemodialysis patients. J Am Soc Nephrol.

[21] Federal.Joint.Committee (2008) Transactions of the German Federal Ministries. BAnz 138:3321

